# Key Assumptions Underlying a Competency-Based Approach to Medical Sciences Education, and Their Applicability to Veterinary Medical Education

**DOI:** 10.3389/fvets.2021.688457

**Published:** 2021-06-02

**Authors:** Jared A. Danielson

**Affiliations:** College of Veterinary Medicine, Iowa State University, Ames, IA, United States

**Keywords:** competency based veterinary education, veterinary medical education, programmatic assessment, competence, outcomes-based education

## Abstract

This perspective explores six key assumptions of a competency-based approach to medical-sciences education, as they relate to veterinary medical education. Those assumptions, derived from characteristics of competency based medical education (CBME) identified by CBME proponents are: (1) There are sufficient shortcomings in the medical competence of graduate veterinarians that solutions are necessary, and changes in the way we teach veterinarians will address those problems. (2) It is feasible to identify generally accepted core competencies in veterinary medical practice. (3) Teaching to defined learning outcomes will produce greater achievement for learners than approaches that do not emphasize clearly defined outcomes. (4) In veterinary medical education, it is possible to articulate the development of competence sequentially in a manner that is relatively consistent across learners, and carefully planning and sequencing learning activities will produce better learning outcomes. (5) Competency-focused instruction, which tailors the pace and progression of instruction to learners, is feasible in veterinary medical education, and will produce better outcomes than instruction that moves all students through an equivalent process in a set time frame. (6) Programmatic Assessment, including numerous direct observations with feedback, will improve learning outcomes, and is feasible in veterinary medical education. While available research does not unequivocally support all six assumptions, overall the potential benefits of adopting a competency-based approach seem promising for veterinary medical education.

## Introduction

Competency based veterinary education (CBVE) is a relatively recent movement in veterinary medicine (1, 2) that borrows from competency based medical education (CBME). CBME, originally introduced in 1978 (3), has gained prominence in the last several decades (4). The purpose of the present paper is to discuss common assumptions associated with competency-based medical education, from the perspective of proponents of that approach, and explore implications of those assumptions as they apply to veterinary medical education.

Numerous researchers have characterized CBME using a variety of approaches and perspectives [e.g., (4–8)], and a thorough review of such efforts is beyond the scope of this paper. In the present discussion, three papers were selected to characterize underlying assumptions of CBME from the perspective of its proponents. Frank and colleagues' *Competency-based medical education: theory to practice* (4) was chosen as an early effort to define the CBME paradigm from the perspective of an international panel of CBME experts. Similarly, Van Melle et al. s' *Core Components Framework* (4) was chosen as a more current synthesis of expert perspectives regarding CBME. A third paper, Holmboe et al. s' *A call to action*. (7) defines CBME in the context of defending against current criticisms of CBME. These papers were selected as broadly representing CBME from the perspective of its proponents, with two of them specifically representing a consensus view, and one specifically addressing criticisms of CBME. Studies exploring implementations of CBME, such as Brydges et al. s' 2021 critical narrative review (5), were not included in this discussion. Such research, while highly relevant to CBME, captures practices that go beyond proponents' recommendations, and therefore beyond the focus of the present discussion.

Table 1 contains the key characteristics identified by Frank et al. (4), Holmboe et al. (7), and Van Melle et al. (8) (Columns 2–4). The first column contains key assumptions proposed herein to underlie the elements of each characteristic. The proposed list of key assumptions derived from the characteristics of competency based medical education and applied to veterinary medical education are as follows:

1. There are sufficient shortcomings in the medical competence of graduate veterinarians that solutions are necessary, and changes in the way we teach veterinarians will address those problems.
2. It is feasible to identify generally accepted core competencies in veterinary medical practice.
3. Teaching to defined learning outcomes will produce greater achievement for learners than approaches that do not emphasize clearly defined outcomes.
4. In veterinary medical education, it is possible to articulate the development of competence sequentially in a manner that is relatively consistent across learners, and carefully planning and sequencing learning activities will produce better learning outcomes.
5. Competency-focused instruction, which tailors the pace and progression of instruction to learners, is feasible in veterinary medical education, and will produce better outcomes than instruction that moves all students through an equivalent process in a set time frame.
6. Programmatic assessment, including numerous direct observations with feedback, will improve learning outcomes, and is feasible in veterinary medical education.

**Table 1:** Underlying assumptions of a competency based approach and implications for instruction, assessment, and feasibility tied to characteristics of CBME as identified by Frank et al. s' (4) *Competency-based medical education: theory to practice*, Holmboe et al. s' (7) *A call to action*, and Van Melle et al. s' (8) *A core components framework*. (2019).

**Assumptions**	**Characteristics of CBME per Frank et al. *CBME: theory to practice***	**Characteristics of CBME per Holmboe et al. *A call to action***	**Characteristics of CBME per VanMelle et al. *A core components framework***
1. There are sufficient shortcomings in the medical competence of graduate veterinarians that solutions are necessary, and changes in the way we teach veterinarians will address those problems. 2. It is feasible to identify generally accepted core competencies in veterinary medical practice. 3. Teaching to defined learning outcomes will produce greater achievement for learners than approaches that do not emphasize clearly defined outcomes.	A focus on curricular outcomes	Graduate outcomes in the form of achievement of predefined desired competencies are the goals of CBME initiatives. These are aligned with the roles graduates will play in the next stage of their careers.Predefined competencies are derived from the needs of patients, learners, and institutions and are organized into a coherent guiding framework (e.g. CanMEDS 2015, ACGME Clinical Competencies).	Clear articulation of outcome competencies
4. In veterinary medical education, it is possible to articulate the development of competence sequentially in a manner that is relatively consistent across learners, and carefully planning and sequencing learning activities will produce better learning outcomes.	An emphasis on abilities (a hierarchy of competencies as the organizing principle of curricula)	Teaching and learning experiences are sequenced to facilitate an explicitly defined progression of ability in stages.	Progressive sequencing of competencies and their developmental markers
5. Competency-focused instruction, which tailors the pace and progression of instruction to learners, is feasible in veterinary medical education, and will produce better outcomes than instruction that moves all students through an equivalent process in a set time frame.	A de-emphasis of time-based training The promotion of learner-centredness	Time is a resource for learning, not the basis of progression of competence (e.g. time spent on a ward is not the marker of achievement).Learning is tailored in some manner to each learner's progression.	Competency-Focused instruction Tailored learning experiences
6. Programmatic Assessment, including numerous direct observations with feedback, will improve learning outcomes, and is feasible in veterinary medical education.		Numerous direct observations and focused feedback contribute to effective learner development of expertise. Assessment is planned, systematic, systemic, and integrative.	Programmatic Assessment

The theoretical assumptions and accompanying recommended practices of a competency-based approach are not entirely independent of each other. For instance, “tailoring learning” to an individual (column 3, row 3) implies “numerous direct observations with feedback” (column 3, row 4), because instruction cannot be tailored to individuals without knowledge of their individual knowledge and skills. Therefore, understanding that some overlap will naturally exist, each row in columns 2–4 contains the characteristic of outcomes-based instruction from each list that seem most closely related to each other.

The following discussion reviews each proposed underlying assumption, identifying broad areas of research relating to each assumption, and discusses challenges and opportunities specific to veterinary medical education. A vast body of research could be consulted to examine these assumptions, however a thorough review of all relevant literature, either within medical education or beyond it, is well-beyond the scope of this paper. Therefore, this discussion relies heavily on meta-analyses such as John Hattie's second order meta-analysis (9) of factors influencing achievement across multiple populations of learners and a variety of learning contexts, as well as meta-analyses specific to medical sciences education and other selected studies where appropriate.

## The Assumptions

### There Are Sufficient Shortcomings in the Medical Competence of Graduate Veterinarians That Solutions Are Necessary, and Changes in the Way We Teach Veterinarians Will Address Those Problems

Well-documented concerns with the performance of recently graduated physicians has been a considerable driver of CBME (7, 10–12). Much less research is available to document the competence of veterinary graduates, though the sparse available evidence suggests that many new veterinarians are not fully prepared for Day 1 practice. Duijn et al. (13) found that veterinary graduates in the Netherlands had practiced for 10 months before they felt fully competent in all five measured outcomes. Similarly, a 2003 survey of members of the Canadian Veterinary Medical Association revealed that 76% of respondents found competence and/or confidence of new graduates to be a moderately (62%) or extremely (14%) serious problem (14). It seems unlikely that these are isolated findings. Unlike human medicine, however, there is no evidence of a broad public perception of a deficiency in veterinary proficiency in the nations represented by the English language literature. In a nationally representative sample of respondents in the USA, Kedrowicz and Royal (15) found a significantly higher opinion of veterinarians than physicians, including in characteristics such as skill, competence, thoroughness, helpfulness, respect, and caring. Similar public sentiments have been identified in the UK and Canada (16, 17). Of course, there are many contextual factors independent of actual competence that affect public perception, but the fact remains that perception of veterinarians is positive.

If a deficiency in new veterinary graduates has not been established, there is no pressing need to prove that adopting CBVE would address that problem. However, if there were such a need, doing so would be a tall order. Researchers in human medical education have found it stubbornly difficult to establish clear relationships between adopting CBME and the resulting competence of medical graduates (5, 18) though available evidence does suggest that it frequently improves patient care (5). The lack of clear research findings is not surprising given the complexity of the research task. Many factors other than educational approach can influence subsequent physician performance, including economic incentives/disincentives, individual factors and the admissions policies that select for them, government and institutional regulation/policy, and accreditation requirements. The problem of establishing a causal link between education and subsequent proficiency in veterinary medicine is, if anything, more difficult than it is in human medicine, because the domain of veterinary practice lacks many of the standardized and readily available dependent measures found in human medicine.

Ultimately, therefore, lacking a clear societal call for change or unambiguous direct evidence that adopting CBVE will influence practitioner competence, advocates for a competency-based approach must, at present, look elsewhere for support for CBVE.

### It Is Feasible to Identify Generally Accepted Core Competencies in Veterinary Medical Practice

The most fundamental and frequently problematic assumption of an outcomes-based approach is that stakeholders can identify and agree upon desired outcomes. A common concern is that essential current or as yet unimagined future competencies may be excluded. While much broader in scope, Bloom's Taxonomy, which sought to provide a conceptual framework by which any learning outcome could accurately be classified, illustrates the challenge (19). Bloom's taxonomy was criticized on a number of grounds, including that there were critical learning outcomes that it could not be used to classify (20). A more recent effort to implement an outcomes-based approach, the “No Child Left Behind” (NCLB) law, adopted in the United States in 2002, illustrated a similar phenomenon. NCLB produced improvement in targeted areas, but was widely criticized (and ultimately not reauthorized) in part because student achievement in outcome areas not emphasized by NCLB, such as music and the arts, suffered from neglect (21). Concerns with CBME have similarly often focused on what is left out—part of the criticism that CBME is reductionist (7).

Anecdotal/unpublished concerns regarding the CBVE framework (22) have frequently also focused on seemingly essential outcomes that are perceived to be left out, or underemphasized. This phenomenon seems inevitable, and, therefore, will almost certainly continue to affect efforts to establish CBVE. Notwithstanding the challenges inherent in identifying core competencies, several factors may improve the likelihood of broad acceptance of a core competency framework in veterinary medical education. First, as is the case with other accredited disciplines, accreditation standards [e.g., (23)] have already produced a level of uniformity that provides common ground for accredited colleges. Second, the high demand for social accountability that accompanies medical practice provides impetus for a competency-based approach; high-stakes outcomes play out daily in clinics and laboratories, providing a constant reminder of the need for competence. Third, and uniquely, the veterinary medical education community is relatively small. Unlike Bloom and colleagues, who sought to account for any learning outcome imaginable, or the authors of the NCLB, who sought to meet the needs of every child in the United States, or even CBME advocates, who seek to satisfy stakeholders in the expansive realm of medical education, a CBVE outcomes framework would serve a relatively modest number of stakeholders. The task of agreeing on outcomes, while daunting, should be more modest that it would be in many other disciplines. The CBVE competency framework (22) has been offered as a place to start, and may help to catalyze further discussion and, potentially, agreement.

### Teaching to Defined Learning Outcomes Will Produce Greater Achievement for Learners Than Approaches That Do Not Emphasize Clearly Defined Outcomes

There is ample evidence to suggest a robust relationship between a focus on defined learning outcomes and improved achievement. Hattie (9) identified 11 meta-analyses involving 604 studies, 41,342 study participants and 820 effects of the use of learning goals to influence subsequent learner achievement. The overall effect of using learning goals was 0.56, larger than the 0.40 effect size considered a meaningful or “desired effect” by Hattie, and the 34^th^ largest effect of the 138 effects he identified. Not all goals provide an equivalent effect. Hattie reported that to be most effective, goals should be appropriately challenging, clear, and associated with clear learning intentions and success criteria.

Specifically informing learners of learning intentions, as well as what constitutes success was also shown across multiple studies to improve achievement. In a subsequent review of meta-analyses with a specific focus on higher education, Hattie identified two of six key findings that that relate directly to explicit outcomes, including “When teachers explicitly inform the students about what success looks like near the start of a series of lessons” (*d* = 0.77) and “When teachers set appropriate levels of challenge.” (*d* = 0.57) [(24), p. 81].

Therefore, overall, this assumption of a competency-based approach is well-supported. Assuming that veterinary educators employ them effectively, the use of carefully defined learning outcomes would be likely to bolster subsequent achievement in veterinary medical education.

### In Veterinary Medical Education, It Is Possible to Articulate the Development of Competence Sequentially in a Manner That Is Relatively Consistent Across Learners, and Carefully Planning and Sequencing Learning Activities Will Produce Better Learning Outcomes

In making their case for including this element of Competency Based Education, Van Melle et al. write “Described as the need to arrange competencies as a ‘sequential path through the programme' while still allowing for considerable flexibility in individual learner progression, mastery learning is a fundamental conceptual framework illuminating how CBME is supposed to work. Accordingly, the sequenced progression of competencies was identified as a core component.” (8) (p. 1005–1006). Certainly mastery learning (discussed more as part of the fifth assumption below), is built on the assumption that learners should not progress to new knowledge until prerequisite knowledge has been mastered, implying, therefore a necessary sequence. As a model, mastery learning has been very effective for promoting achievement both generally (9) and in medical sciences education [e.g., (25)].

It is important to note that this assumption calls for a thoughtful and effective progression of competencies rather than a single immutable curricular sequence. A substantial variation in sequence, such as that seen among disciplines-based, organ-based, and problem based learning curricula have been shown to have a negligible effect on learner achievement (26).

In terms of practicality for veterinary medical education, this particular assumption seems no more difficult than it would for other disciplines. Furthermore, other than conducting a task analysis, it is no more onerous to sequence learning activities effectively than to sequence them ineffectively. The more difficult assumption, that the learning pace can be customized, is discussed as part of the next assumption.

### Competency-Focused Instruction, Which Tailors the Pace and Progression of Instruction to Learners, Is Feasible in Veterinary Medical Education, and Will Produce Better Outcomes Than Instruction That Moves All Students Through an Equivalent Process in a Set Time Frame

Advocates for CBME hold that instruction should focus on the achievement of competence, rather than the passage of time. Ideally, under this assumption, students could complete their veterinary education in however much time was required to achieve competence, taking as much time as needed for difficult sections, and moving as quickly as they desired through less challenging material. Mastery learning (27), perhaps the best known application of this approach, provides learners with a clear understanding of what constitutes mastery, opportunities to practice in a supportive environment, high levels of feedback, and opportunities to continue to review and practice material until mastery is achieved (9). Research suggests that this approach is effective. In seven meta-analyses involving 377 studies, 296 effects, and 9,323 participants, the overall effect size of mastery learning on achievement was found to be 0.58, the 29^th^ highest effect identified by Hattie in his 2^nd^ order meta-analysis (9).

However, despite theoretical advantages, there are substantial pragmatic barriers to allowing students to progress through a veterinary medical education program at their own pace. Such barriers include (a) the need for adapted assessment frameworks (28), (b) complexities in processes such as registration, financial aid, and transcript creation, (c) a loss of economies of scale involving resources such as expensive and limited supplies and specimens, specialized space, and limited instructor time, and (d) challenges in building a sense of community (29). Such constraints have hampered the adoption of a competency rather than time-based approach in human medical education, and they are likely to pose a similar, if not greater barrier for veterinary colleges, which tend to operate with more limited resources than human medical colleges.

Other potential elements of a competency-based instructional approach that tailor instruction to the individual are also resource intensive, but perhaps more achievable for veterinary educators. Frequently assessing progress and providing feedback have been shown to be powerfully effective (9, 24), and are aspects of a CBVE approach that should prove beneficial. Furthermore, while it can be time consuming to measure progress, provide regular feedback, and adapt instruction to instructional needs diagnosed at the individual or course level, advances in instructional technology and technology-enhanced teaching methodologies are increasingly making such approaches feasible. *Team Based Learning* (TBL), for instance, which aims to make effective pedagogical approaches such as frequent practice with immediate feedback feasible in a large classroom, has been shown to be effective in human medical education (30, 31).

### Programmatic Assessment Will Improve Learning Outcomes, and Is Feasible in Veterinary Medical Education

Van Der Vleuten et al. (32) characterize programmatic assessment as an “assessment as learning” approach “where assessment of and for learning are merged” (p. 621). In this approach, high stakes assessment decisions are based on many individual data points involving assessments ranging from low to high stakes, involving a variety of methods, occurring over time, and always providing meaningful feedback to learners. Research suggest that this approach is promising both for assessment and for learning. With its emphasis on frequent, specific feedback, and the use of formative evaluation, this approach finds strong support in a large volume of research emphasizing the usefulness of both formative evaluation and feedback-guided practice and learning (9, 24, 33, 34). Additionally, while still few in number, existing studies of the impact of this approach on student achievement in medical education are promising (35). In a veterinary education setting, Bok et al. found strong validity evidence for a programmatic assessment approach (36), suggesting that while leveraging the benefits of improved learning, institutions can also improve their knowledge of learner improvement and achievement.

## Criticisms of a Competency-Based Approach

It is important to acknowledge criticisms of competency-based approaches. In summarizing these criticisms, Holmboe et al. (7) identified five broad areas of concern, including fears of reductionism, a lack of evidence for CBME, conceptual and pragmatic challenges with a competency rather than a time-based focus, challenges with implementing CBME, and other philosophical/ideological concerns. Philosophical concerns, such as the concern with reductionism, echo a much older tension among objectivist, interpretivist and pragmatic epistemologies (37), and highlight the challenges in finding common theoretical ground. Such tensions seem just as likely in veterinary medical education as in similar educational disciplines. Similarly, as noted in prior sections, the pragmatic concerns encountered in medical education generally seem likely to pose similar challenges to veterinary educators. A detailed exploration of each such criticism is beyond the scope of this discussion. Rather, this discussion seeks to explore the assumptions underlying a competency-based approach, thereby helping inform decisions regarding whether the potential benefits of adopting CBVE are likely to justify the inevitable challenges.

## Conclusion

A theoretical exploration of assumptions underlying a competency-based approach to medical education highlight a number of potential advantages, as well as some challenges that might be heighted in veterinary education. Unlike in human medical education, in veterinary medical education, social perceptions of veterinary competence are not an important driver of a competency-based approach. Compared to human medical education, veterinary medical education is a relatively smaller community, with fewer resources. This provides an advantage when considering adopting CBVE, because fewer stakeholders decrease the complexity of arriving at a consensus regarding what constitutes competence. At the same time, however, fewer resources reduce the practicality of resource-intensive aspects of CBVE, such as using a competency rather than time-focused instructional process. Complexities aside, many aspects of a CBVE approach, including identifying and teaching to defined learning outcomes, a focus on mastery, and the use of programmatic assessment, have all been shown to be practical and effective in medical-education settings. Overall, the potential benefits of a competency-based approach seem sufficiently promising to recommend it, notwithstanding the concerns and complexities that inevitably arise.

## Data Availability Statement

The original contributions presented in the study are included in the article/supplementary material, further inquiries can be directed to the corresponding author/s.

## Author Contributions

The author confirms being the sole contributor of this work and has approved it for publication.

## Conflict of Interest

The author declares that the research was conducted in the absence of any commercial or financial relationships that could be construed as a potential conflict of interest.
